# Quantitative evaluation of canine urinary bladder transitional cell carcinoma using contrast-enhanced ultrasonography

**DOI:** 10.1186/s12917-018-1384-5

**Published:** 2018-03-12

**Authors:** Francesco Macrì, Simona Di Pietro, Cyndi Mangano, Michela Pugliese, Giuseppe Mazzullo, Nicola M. Iannelli, Vito Angileri, Simona Morabito, Massimo De Majo

**Affiliations:** Department of Veterinary Sciences, University of Messina, Polo Universitario Annunziata, 98168 Messina, Italy

**Keywords:** Dog, Contrast-enhanced ultrasonography, Diagnostic ultrasound, Urinary bladder, Sonovue, Qontrast

## Abstract

**Background:**

In veterinary medicine, contrast-enhanced ultrasonography allowed the accurate quantification of liver, splenic and kidney vascularization in healthy dogs and the differentiation between malignant and benign hepatic, renal, and splenic nodules in dogs and cats based on perfusion patterns. The utility of contrast-enhanced ultrasonography in other applications is still under study.

The aim of this study was to develop diagnostic criteria by contrast-enhanced ultrasonography in 8 client-owned adult dogs affected by urinary bladder transitional cell carcinoma with definitive diagnosis made by cytopathologic evaluation after suction biopsy. The contrast enhancement pattern and the quantification of blood flow parameters of this tumor were reported.

**Results:**

Examinations with B-mode, Doppler ultrasonography and contrast-enhanced ultrasonography were performed in all not sedated dogs. Assessments of bladder masses and bladder wall infiltration were performed. Each dog received 2 bolus injections of sulfur hexafluoride during the contrast-enhanced ultrasonography. Quantitative analysis of the contrast-enhanced ultrasonography images were performed. For each dog, one region of interest was manually drawn around the entire tumor. Software analysis of contrast-enhanced time-intensity curves was used to identify peak enhancement, time to peak enhancement, regional blood volume, regional blood flow, and mean transit time.

Contrast-enhanced ultrasonography showed an avid enhancement of the tumour tissue, with a heterogeneous or homogeneous pattern. The exam also showed the loss of planes between the lesion and the muscular layer. The presence of vascularized tissue through the bladder wall confirms the infiltrative feature of the tumour. Post-processing quantitative analysis showed a time-intensity curve with a rapid wash-in, a low level of signal intensity and a slow wash-out.

**Conclusions:**

Contrast-enhanced ultrasonography provided useful clinical information and defined a vascular enhancement patterns and calculated parameters associated with TCC. It may be a useful, noninvasive and reproducible tool for detecting these tumors in dogs.

## Background

Contrast-enhanced ultrasound is a technique that involves the use of ultrasound contrast agents to improve the traditional ultrasonography both in human and veterinary medicine [1, 2].

Contrast agents used in CEUS are composed of gas-filled microbubbles, which are injected into the bloodstream. When the power of the ultrasound beam is low enough, microbubbles resonate, rapidly contracting and expanding in response to the pressure changes of the sound wave. Multiple harmonic signals are produced that can be recorded [3].

Following injection, the bubbles greatly increase the amplitude of the scattered signals not only from large vessels but also from the microvasculature, providing real-time assessment of vascular perfusion of target organs and/or lesions [4]. Contrast media are safe products and are well tolerated by dogs and cats [5].

In veterinary medicine, CEUS allowed the accurate quantification of liver, splenic and kidney vascularization in healthy dogs [6–8] and the differentiation between malignant and benign hepatic, renal, and splenic nodules in dogs and cats based on perfusion patterns [9–13].

The utility of CEUS in other applications is still under study. Recently, the use of CEUS has been extended to imaging of the urinary bladder in humans, improving the differential diagnosis between urinary bladder intraluminal masses and clots and allowing the differentiation between enhancing and nonenhancing lesions [14, 15].

Some Authors also demonstrated that it is possible to predict the human bladder neoplasm grading by the pattern of CEUS time–intensity curves [16, 17].

Contrast-enhanced ultrasound also resulted to be superior to grayscale ultrasound in assessing infiltration of the muscle wall-layer [3].

In veterinary medicine, urinary bladder tumors affects commonly dogs and the most common cancer of the canine urinary bladder is the invasive TCC of intermediate to high grade.

A study reported ultrasonographic findings such as wall involvement, heterogeneous mass and trigone location could be reliable prognostic indicators in canine TCC [18].

To our knowledge, there are no published reports using CEUS in the diagnosis of urinary bladder diseases in dogs.

In this study we describe the application of qualitative and quantitative CEUS in eight dogs affected by bladder TCC, reporting the contrast enhancement pattern and the quantification of blood flow parameters of this tumor, in order to provide a useful diagnostic tool for a more complete evaluation of the canine bladder TCC.

We hypothesized that CEUS would improve the detection of the infiltrative feature of this tumor by observing the abnormal intramural perfusion pattern.

## Methods

### Study population

To test our hypothesis, over a year’s time (October 2015, November 2016) we recruited 8 consecutive dogs with cytopathologic diagnosis of TCC based on suction biopsy as “gold standard”.

The cytological examination, reviewed by a veterinary pathologist (GM), showed scarce basophilic background, sometimes strongly hematic. Cells were represented by two types of epithelial elements. A first type consisted of single or small groups of epithelial elements in dysplastic squamous features. The second type was represented by more or less large epithelial aggregates with basophilic, vacuolated cytoplasm and rounded margins. Nuclei were single, sometimes multiple, often bulky, irregularly shaped, with angled profile or indented and abundant chromatin disposed in granules or large clusters. Anisocytosis, anisokaryosis and pleomorphism were evident. N/C ratio > 1 and the mitotic index was moderate. The findings were strongly suggestive of a cytological diagnosis of transitional cell carcinoma.

There were 3 spayed female, 2 entire female, 3 entire males. There was 1 each Saint-Bernard, Spinone Italiano dog, Cocker, Collie, Beagle and 3 mixed breed. The mean (± SD) age of the eight enrolled dogs was 6.8 (± 2.6) years (range: 3 to 10 y).

Dogs were excluded, if they had evidence of cardiac disease or a history of anaphylactic reactions to vaccines or other medications, to avoid adverse reactions due to the microbubble contrast agent. Dogs were also excluded if their complete blood count and serum biochemistry were not within normal range.

### B-mode and color Doppler ultrasonography

Examinations with B-mode, Doppler ultrasonography and CEUS were performed on all dogs.

Informed owners’ consents were obtained. All treatments, housing, and animal care were in compliance with EU Directive 2010/63/EU on the protection of animals used for scientific purposes and with Department’s Animal Ethics Council approval (no. 13/2017).

The conventional ultrasonography and the CEUS examinations were performed by the same investigator, using a scanner^1^ equipped with contrast-tuned imaging technology^2^ in not sedated dogs in dorsal recumbency. Hairs over the ventral portion of the abdomen were clipped. Alcohol and coupling gel were applied to the skin.

B-mode and Color Doppler ultrasonography of bladder was performed with microconvex (5.0 to 8.0-MHz) and linear (10 to 12-MHz) transducers. Transverse and longitudinal planes were used to fully assess the bladder.

Ultrasonographic assessments of bladder masses included: size and shape (pedunculated or nonpedunculated), echo pattern (homogeneous or heterogeneous), location (apex, body, trigone or all sites), and involvement of the bladder wall. Bladder masses having uniform echogenicity or mixed echogenicity with hyperechoic and hypoechoic interior areas were recorded as “homogeneous” or “heterogeneous”, respectively.

The main diagnostic criterion used to diagnose tumor infiltration of the bladder wall was the interruption of hyperechoic (submucosal) and hypoechoic (muscular) layers of the bladder wall in the site of the lesion, as described in previous reports [18].

### Contrast-enhanced ultrasonography

CEUS examination was performed immediately following B-mode ultrasonography, using a linear (5.0-7.5-MHz) transducer with contrast agent capability. The mechanical index was set from 0.08 to 0.09; only 1 focal zone was used, which was placed immediately below the urinary bladder.

The contrast agent was a sulphur hexafluoride signal enhancer^3^ and it was prepared in accordance with the manufacturer’s recommendations. Each vial of contrast agent (which contained 25 mg of freeze-dried powder) was reconstituted by injection of 5 mL of 0.9% sodium chloride; vials then were shaken vigorously for 20 s. An aliquot (0.05 mL/kg of body weight) of the contrast medium was rapidly injected via a 3-way valve and 18-20 G catheter inserted in the cephalic vein. The contrast injection was immediately followed by a 5 mL saline flush, as previously reported [13].

Each dog received 2 bolus injections of contrast agent; the second injection of ultrasound contrast agent was 10 min later the first. The activation of a timer was performed simultaneously with the contrast agent dose inoculation.

All CEUS examinations were performed by two operators. The first operator injected the contrast medium through the catheterized vein, while the second performed the US scans of the urinary bladder.

Raw data (good-quality video clips) obtained during CEUS were stored digitally on a hard disk and subsequently they were analysed by two co-authors.

Post processing quantitative analysis of video-clips was performed by use of image-analysis software.^4^ For each dog, one region of interest (ROI) was manually drawn around the entire tumor. During ROI selection adjacent portions of the bladder wall that appeared unaffected were excluded.

Contrast-enhanced TICs were generated for each ROI. Analysis of tissue perfusion was based on video SI changes over time using CEUS.

The SI of a white band in the gray scale bar (8 bit) was defined as maximal (100%) SI. Other pixels in the image were then assigned SI values based on this reference.

Within the selected ROI, during the period of enhancement, the following parameters were computed: peak enhancement, TTP perfusion, MTT, RBV and RBF. Peak enhancement (%) is defined as the percentage increase in SI - from 0 to 100 as maximal intensity - reached during transit of the contrast agent at a specific time point. The TTP (seconds) is defined as the interval until maximum SI of the contrast agent. The MTT is defined as the circulation time of the contrast agent in the examined tissue. The RBV is defined as the integral of the video SI (%) changes during the extrapolated transition time without recirculation. The RBF is defined as the ratio between regional blood volume and MTT.

A three-dimensional color map was reconstructed to visually assess regional blood flow of the corresponding bladder tumor.

Descriptive statistical analysis of quantitative CEUS-derived data included Shapiro-Wilk test, assessing the normal distribution of variables. Obtained data are expressed as mean ± standard deviation.

## Results

**Fig. 1:**
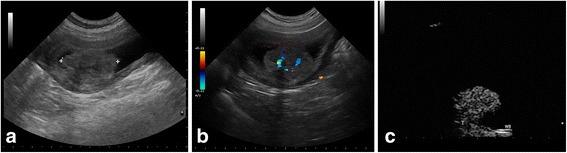
6 year-old entire male Spinone Italiano dog with pedunculated round shaped mass, located at the level of the bladder apex, staged with gray-scale ultrasound and CEUS. **a**- Conventional B-mode sonography shows the bladder mass with homogeneous echo pattern and interruption of hyperechoic bladder wall by less echogenic tumor tissue. **b** – Colour Doppler shows the presence of discrete vascular poles within the mass. **c** - CEUS image shows the avid homogenous enhancement of tumour tissue and the loss of bladder wall hypoenhancing muscular layer at base of tumor due to the appearance of hyperenhancing tumor tissue (T). In the normal wall bladder (WB) adjacent to the tumour the submucosal and serous hyperenhancing lines and the hypoenhancing muscular layer were preserved

Trans-abdominal grey-scale ultrasonography, performed on dog 1, revealed the presence of a large non pedunculated cauliflower shaped mass (40 × 40 mm), located at the level of the entire dorsal wall of the bladder and protruding into the lumen. Furthermore, it had jagged margins. The echo pattern of the mass was heterogeneous. Colour Doppler showed an abundant vascularization with more than one vascular pole (Fig. 1a and b).

**Fig. 2:**
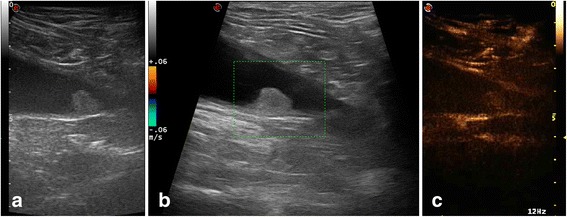
9 year-old entire female mixed breed dog with nonpedunculated round shaped mass, located at the level of the bladder trigone. B-mode ultrasonography shows the bladder mass with homogeneous echo pattern and interruption of hyperechoic bladder wall by less echogenic tumor tissue (**a**), without evidence of vascular signal (**b**). CEUS image shows the avid homogenous enhancement of tumour tissue and the loss of bladder wall hypoenhancing muscular layer at base of tumor (**c**)

**Fig. 3:**
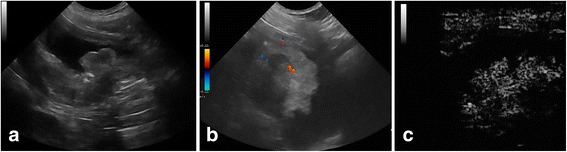
3 year-old entire male Saint-Bernard dog with nonpedunculated cauliflower shaped mass, located at the level of the bladder body. **a**- Conventional B-mode sonography shows the bladder mass with heterogeneous echo pattern and interruption of hyperechoic bladder wall by less echogenic tumor tissue. **b** – Colour Doppler shows the presence of discrete vascular poles within the mass. **c** - CEUS image shows a heterogeneous enhancement of tumour tissue and the loss of bladder wall hypoenhancing muscular layer at base of tumor due to the appearance of hyperenhancing tumor tissue

In all other dogs pedunculated round shaped masses of variable size from 10 to 30 mm, located at the level of the trigone (4 cases) and apex (3 case) of the urinary bladder, protruding into the lumen were observed. Conventional B-mode and Doppler ultrasonography showed different patterns of tumors (Figs. 2a, b, 3a, b).

In all cases, the normal bladder wall was identified by ultrasound as two hyperechoic parallel thin layers and a hypoechoic layer that corresponds to the muscular layer, placed between the two previous ones. The portion of bladder wall located at base of tumor showed the loss of normal layering, that induced the suspicion of an intramural neoplastic invasion.

Contrast-enhanced ultrasound images showed in the arterial phase, at 8-9 s after the contrast injection, an avid enhancement of the tumour tissue in all dogs, with a heterogeneous enhancement in dog no. 1 and no. 3 (Figs. 1c and 3c) for the presence of several small non-enhancing areas into the contest lesion consistent with necrotic lesions, and with a homogeneous enhancement of masses in all other dogs (Fig. 2c).

Contrast-enhanced ultrasound images also showed the loss of planes between the lesion and the bladder wall layers, with disappeared hyperenhancing submucosal layer and hypoenhancing muscular layer, due to the presence of the hyperenhancing tumor tissue. The disruption of the bladder wall muscular layer by enhancing tumor tissue confirms the infiltrative feature of tumour (Figs. 1c, 2c and 3c).

In the venous phase there was a slow and progressive reduction of enhancement in the tumour tissue.

Enhancement of the normal wall bladder adjacent to the tumour is almost imperceptible, throughout the arterial and venous phases. During the initial arterial phase, the bladder mural vascularization was characterized by the first appearance of the external layer, followed by small vessels perpendicular to the axis of the wall, into the hypoenhancing muscular layer, that appeared approximately 11 s after the contrast agent injection. About 22 s from the contrast dose, the submucosal layer became progressively enhanced. In the normal portions of the wall bladder the submucosal and serous hyperecoic lines were preserved (Figs. 1c, 2c, 3c).

**Fig. 4:**
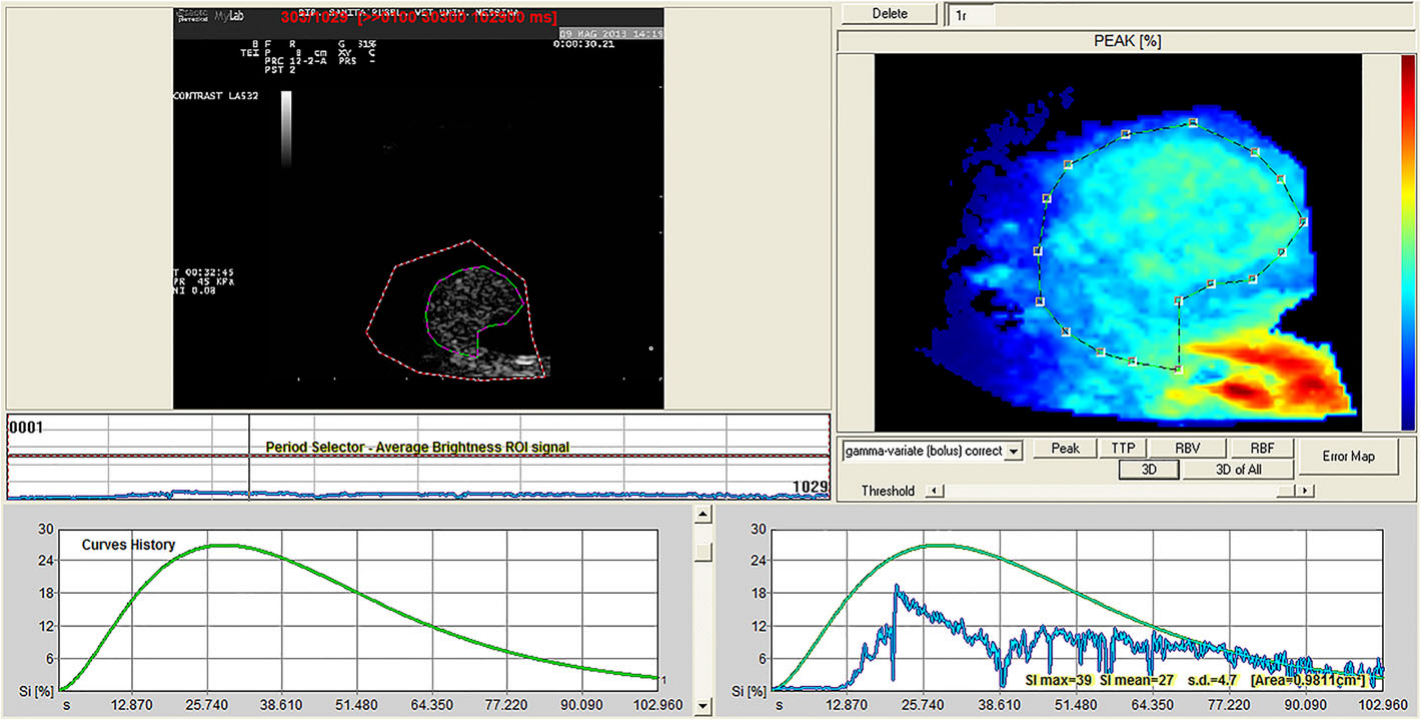
Quantitative analyses performed after injection of contrast agent with dedicated perfusion software. Time-intensity curve was created from the ROI positioned in the tumor tissue of a representative dog with urinary bladder TCC. The jagged gray line was generated from actual data, whereas the curved black line was generated from data fitted by use of a corrected γ function

Post-processing quantitative analysis showed a time intensity curve characterized by a rapid wash-in, a low level of SI and a slow wash-out (Fig. 4).

**Fig. 5:**
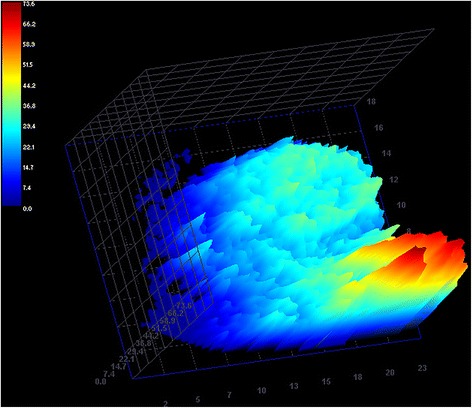
Three-dimensional CEUS reconstruction demonstrating regional blood flow of the tumor tissue during the wash-in phase in a dog with urinary bladder TCC, expressed in arbitrary units of signal intensity. Note the homogeneous perfusion pattern of the tumor tissue

The three-dimensional images of regional blood flow underlined a homogeneous perfusion patterns of the tumor tissue, as well as observed during the qualitative analysis, providing a more intuitive reconstruction of vascular features that characterized the urinary bladder TCC (Fig. 5).

Descriptive statistical analysis of the quantitative CEUS-derived parameters revealed a normal distribution for each value of its mean and standard deviation, provided below: peak enhancement was 22,05 ± 1,73%; TTP was 30,38 ± 4,78 s; RBV was 1064.15 ± 184,39 L/min; RBF was 25,17 ± 3.29 L/min; MTT was 42,19 ± 3,81 s.

In all cases the contrast micro-bubbles were well tolerated by dogs and no side effects were noted.

## Discussion

Transitional Cell Carcinoma is the most common malignancy of the urinary bladder in dogs (50-75%), generally invasive and often metastatic to the lung, regional lymph nodes or liver [19, 20]. Multiple risk factors for the development of canine TCC are involved, including both genetic and environmental factors. A breed predisposition (Scottish Terrier, Shetland Sheepdog, Collie, Airedale and Beagle) and a major incidence of TCC in older females were reported [19]. The most common clinical sign in dogs is dysuria, including haematuria, abnormally frequent and painful excretion of drop by drop urine, and it can be present for weeks to months prior to definitive diagnosis [21]. This tumor is frequently located in the trigon region of the canine urinary bladder [22–24]; sometimes an urethral involvement, that can lead to dysuria and partial or complete obstruction of the urinary tract, may be seen [25].

It is associated with short-survival time [19, 21]. Due to the progressive nature of this disease, the treatment, including the surgical excision and the combination chemotherapy, is palliative.

Diagnostic methods to detection the TCC include radiography (double contrast cystography) and ultrasound examination. Definitive diagnosis can be achieved through histopathologic examination of a tissue sample obtained by cystotomy, cystoscopy, or a catheter biopsy [22, 24].

In the present study, the diagnostic criteria of the canine TCC were based on a cytopathologic evaluation of a sample of cancerous cells obtained through an ultrasound-guided suction biopsy and smeared on slides and then stained with May-Grümwald Giemsa.

An accurate evaluation of tissue layers of the urinary bladder wall is required for diagnosing and predicting prognosis in transitional cell carcinoma, so that it can be differentiated from other benign lesions [18].

Ultrasonography is commonly used as the first-line imaging modality in order to diagnose a bladder cancer, because it is not invasive, widely available and does not require anesthesia [26]. Furthermore, the detection of tumoral angiogenesis using an imaging technique is very important to achieve a diagnosis of malignant cancers. So, imaging techniques performed with the injection of intravascular contrast agents become the gold standard to fully study an invasive bladder tumors in humans as well as in animals.

Various studies demonstrated that high-field magnetic resonance imaging and computed tomography using iodinate contrast media could be valuable for evaluation of wall involvement in human and canine bladder cancer [20, 27, 28].

However, in clinical practice computed tomography and magnetic resonance imaging scan require anesthesia, are very expensive and therefore not widely available and sometimes unsafe because of adverse reactions associated with the use of iodine as a contrast medium.

So, CEUS may play a role in urinary bladder diagnostic field, being helpful in differentiating pathological entities, as clots or surgical wall changes, from bladder cancer, detecting bladder tumour neovascularisation enhancement [14].

In human medicine, qualitative contrast ultrasound has been used to identify bladder tumors and to evaluate enhancement patterns [14, 29].

Some Authors showed that CEUS had a very high sensitivity for the presence of human bladder cancer and that it is very sensitive in revealing tumor microvascularisation [14].

In veterinary medicine, some Authors reported that in canine urinary bladder lesions wall involvement, as revealed by B-mode ultrasound, was significantly (*P* = 0.00005) associated with histological muscular layer involvement with a sensitivity of 93% and specificity of 92%, showing that this ultrasonographic finding could be reliable indicator of prognosis in canine transitional cell carcinoma [18].

A recent study was carried out to evaluate the feasibility of using contrast-enhanced cadence contrast pulse sequencing ultrasound estimates of microvascular density to predict clinical and angiogenic response to treatment in dogs affected by bladder tumors undergoing to chemotherapy [30].

In our study, using a contrast-tuned imaging technology, CEUS provided useful clinical information and defined a vascular enhancement patterns and calculated parameters associated with TCC.

The purely intravascular contrast agent provide detailed viewing of the microvascular pattern of the tumor lesion and a more accurate reading of ultrasound images.

The qualitative analysis showed a hyperechoic CEUS pattern of the tissue tumor. Previously hypoperfused muscle layer under the mass became hyperenhanced, suggesting that the tumor had infiltrated the bladder wall.

These findings support the use of detailed evaluation of the bladder wall by CEUS for prediction of prognosis in canine TCC, although further studies could be advisable for targeting differential diagnosis among TCC and other proliferative lesions of the canine urinary bladder.

The quantification of blood flow parameters was also reported, although the qualitative analysis of perfusion pattern is more reliable in arriving to a definitive diagnosis. In fact, the ultrasound enhancement measurements may be influenced by several patient-related (i.e. cardiac output, blood pressure, respiratory rate and interactions with the contrast medium) and technical variables related factors (i.e. scanner setting, gain setting, type, preparation and injection protocol of the contrast medium), all of which are potential source of variability.

## Conclusions

In conclusion, our results must be interpreted in light of the relatively small number of dogs enrolled. We believe that this finding should be included in contrast ultrasonography of the canine TCC. CEUS exam may be a useful, noninvasive and reproducible tool for detecting tumors that infiltrate the muscle layer of the bladder wall.

## Abbreviations

CEUS: Contrast-enhanced ultrasonography
MTT: Mean transit time
RBF: Regional blood flow
RBV: Regional blood volume
ROI: Region of interest
SI: Signal intensity
TCC: Transitional cell carcinoma
TIC: Time-intensity curve; TTP: Time to peak

## Footnotes

1 Mylab 40/Vet, Esaote, Genova, Italy.

2 CnTI, Esaote, Genova, Italy.

3 SonoVue®, Bracco Imaging, Milan, Italy.

4 Qontrast™, Bracco, Milan, Italy.
